# Idarubicin-loaded drug-eluting microspheres transarterial chemoembolization for intermediate stage hepatocellular carcinoma: safety, efficacy, and pharmacokinetics

**DOI:** 10.2478/raon-2024-0052

**Published:** 2024-10-04

**Authors:** Spela Korsic, Josko Osredkar, Alojz Smid, Klemen Steblovnik, Mark Popovic, Igor Locatelli, Jurij Trontelj, Peter Popovic

**Affiliations:** Clinical Institute of Radiology, University Medical Centre Ljubljana, Ljubljana, Slovenia; Faculty of Medicine, University of Ljubljana, Ljubljana, Slovenia; Institute of Clinical Chemistry and Biochemistry, University Medical Centre Ljubljana, Ljubljana, Slovenia; Faculty of Pharmacy, University of Ljubljana, Ljubljana, Slovenia; Department of Gastroenterology and Hepatology, University Medical Centre Ljubljana, Ljubljana, Slovenia; Department of Cardiology, University Medical Centre Ljubljana, Ljubljana, Slovenia; Biotechnical Faculty, University of Ljubljana, Ljubljana, Slovenia

**Keywords:** hepatocellular carcinoma, drug-eluting microspheres transarterial chemoembolization, idarubicin, safety, efficacy, pharmacokinetics

## Abstract

**Background:**

Transarterial chemoembolization (TACE) is the treatment of choice for the intermediate stage hepatocellular carcinoma (HCC). Doxorubicin remains the most used chemotherapeutic agent in TACE, although *in vitro* screening has demonstrated that idarubicin exhibits greater cytotoxicity against HCC. This study aimed to evaluate safety, efficacy, and pharmacokinetics of idarubicin-loaded drug-eluting microspheres TACE (DEMIDA-TACE) in intermediate stage HCC patients.

**Patients and methods:**

Between September 2019 and December 2021, 31 consecutive intermediate stage HCC patients (96.8% cirrhotic) were included to this study. 2 mL of LifePearl™ microspheres (100 μm) loaded with 10 mg of 1 mg/mL idarubicin were used for treatment. The adverse events, objective response rate (ORR), progression free survival (PFS), time to TACE untreatable progression (TTUP), median overall survival (mOS), and pharmacokinetics were evaluated.

**Results:**

There were 68 TACE procedures performed. Adverse events grade ≥ 3 were noted after 29.4% procedures. The ORR was 83.9%, median PFS and TTUP were 10.5 months (95% CI: 6.8–14.3 months) and 24.6 months (95% CI: 11.6–37.6 months), respectively. Median OS was 36.0 months (95% CI: 21.1–50.9 months). Significant differences between patients achieving objective response (OR) and those with progressive disease were observed regarding idarubicinol and combined idarubicin-idarubicinol plasma concentrations at 72 hours post-procedure, higher plasma concentrations were observed in patients achieving OR (p = 0.014 and 0.014; cut-off values 1.2 and 1.29 ng/mL, respectively).

**Conclusions:**

DEMIDA-TACE emerges as a safe and effective method of treatment for the intermediate stage HCC with low rates of adverse events alongside high tumor response, favourable disease control and overall survival. Idarubicinol and combined idarubicin-idarubicinol plasma concentrations at 72 hours post-procedure may serve as prognostic factors for achieving OR.

## Introduction

Hepatocellular carcinoma (HCC) is the most frequent primary liver cancer and therefore crucial global medical concern.^1,2^

According to the guidelines of the European Association for the Study of the Liver (EASL), transarterial chemoembolization (TACE) is the treatment of choice for the BCLC-B (Barcelona Clinic Liver Cancer) group, *i.e.* intermediate stage of HCC patients.^3,4^

Despite the wide use of TACE, treatment strategies and procedures are not standardized across different institutions. TACE can be performed as conventional TACE (cTACE) using lipiodol, or with drug-eluting microspheres (DEM-TACE).^3,5^ DEM-TACE allows slow release of chemotherapeutic agents, enhancing the duration and intensity of local ischemia.^6,7,8^ To date, the most used chemotherapeutic agent in TACE is doxorubicin, even though the evidence supporting its use is limited.^9^

In 2011, *in vitro* cytotoxicity screening aimed at selecting the most efficient drug against HCC evaluated 11 most used anticancer drugs. These included anthracyclines (doxorubicin, epirubicin, idarubicin and related mitoxantrone), platinum derivates (cisplatin, carboplatin, and oxaliplatin), antimetabolites (5-flourouracil and gemcitabine), the alkylating antibiotic mitomycin C, and the taxane paclitaxel. Idarubicin proved to be the most toxic drug against HCC *in vitro*, moreover, it also demonstrated efficacy against the resistant SNU-449 cell line where doxorubicin failed to achieve 50% cell death.^9^

Idarubicin is an anthracycline, to date mostly used for the treatment of acute leukaemia, when administrated intravenously. The superior cytotoxicity of idarubicin towards HCC is presumed to result from its high hepatic penetration and highly lipophilic nature compared to other anthracyclines, resulting in high hepatic concentrations. High intracellular concentrations of idarubicin are necessary to achieve its cytotoxicity against multidrug resistance (MDR) mechanisms.^9^ In the liver, idarubicin undergoes reduction to its metabolite idarubicinol, which retains pharmacological activity and is considered equipotent as the parent drug.^10^

Another important feature of idarubicin is its ability to be loaded in drug-eluting microspheres by positively charged protonated amine group of idarubicin hydrochloride interacting with negatively charged sulphonate group of microspheres.^11,12,13^

Moreover, the first results of *in vivo* phase I and II safety and efficacy trials of idarubicin use in cTACE and DEM-TACE have shown promising outcomes in terms of safety profiles and objective response.^12,13,14,15,16^ Therefore, we designed present prospective study to evaluate safety, efficacy, and pharmacokinetics of idarubicin-loaded DEM-TACE (DEMIDA-TACE) for the intermediate stage HCC patients.

## Patients and methods

The study strictly followed the ethical guidelines of the Helsinki Declaration for biomedical research involving human subjects. Approval for the study was obtained from the Republic of Slovenia National Medical Ethics Committee on the 19th of March 2019, with the assigned approval number 0120-64/2019/5.

### Patient selection

Between September 2019 and December 2021, 31 consecutive intermediate stage HCC patients who did not meet exclusion criteria and signed the informed consent were enrolled to this prospective single-institution study. Exclusion criteria included Child-Pugh liver cirrhosis > 8 points, portal vein thrombosis, iodine contrast agent allergy, left ventricular ejection fraction (LVEF) < 50%, acute liver, kidney and/or cardiovascular failure, and women of childbearing age.

The decisions for DEM-TACE and inclusion in the study were reached through weekly multidisiplinary tumour board (MTB) meetings at our institution. The MTB comprises experts in abdominal and interventional radiology, gastroenterology, hepatobiliary surgery, nuclear medicine, and oncology.

The final day of the follow-up for the purpose of present study was December 31, 2023.

### Procedures

All patients received prophylactic antibiotic treatment (*i.e.* 1000 mg of amoxicillin/clavulanic acid) and were scheduled for tumour biopsy in the same session as the first DEMIDA-TACE procedure. Patients were treated using a mixture of 2 mL LifePearl™ (Terumo Europe, Leuven, Belgium) drug-eluting microspheres of 100 μm in diameter loaded with 10 mg of 1 mg/mL idarubicin (Zavedos^®^, Pfizer, France). Superselective approach to treatment with Progreat™ microcatheter (Terumo Europe, Leuven, Belgium) was used in all patients. Before initiating microspheres delivery, a cone-beam computed tomography (CBCT) using Artis Zee floor with DynaCT (Siemens, Forchheim, Germany) was performed to verify the microcatheter's accurate positioning within the feeding artery to ensure a complete coverage of the targeted lesions. For the visualization, a nonionic contrast agent (Ultravist 370^®^, Bayer HealthCare, Germany or Visipaque 320, GE Healthcare, Norway) was administered using a power injector (Avanta^®^, Medrad, Bayer HealthCare, Germany). In patients with multisegmental or bilobar disease, the position of the microcatheter was changed within the same session to ensure superselective delivery to each lesion. The idarubicin-microspheres mixture delivery was slow (1 mL/min) and discontinued after complete dosage administration or at early stasis.

### Adverse events

Safety was evaluated by clinical and laboratory monitoring. Adverse events were assessed according to the Common Terminology Criteria for Adverse Effects v. 5.0 (CTCAE), AEs grade ≥ 3 were noted.^17^ Potential toxicity was assessed in the inpatient setting until the patient's discharge, in the outpatient setting at 72 hours post-procedure, and again after 14 days, as well as with follow-up imaging. Baseline and follow-up peripheral venous blood samples were obtained to assess liver enzymes (alanine aminotransferase [ALT], aspartate aminotransferase [AST], gamma-glutamyl transferase [GGT]), complete blood count (CBC), differential blood count (DBC), coagulation tests, bilirubin, albumin, kidney function (urea, creatinine, estimated glomerular filtration rate [eGFR]) and alpha fetoprotein (AFP).

Before inclusion in the study, all patients underwent a cardiac ultrasound performed by an experienced cardiologist to exclude individuals with a LVEF lower than 50%. For cardiotoxicity evaluation, LVEF was measured at baseline, 1 month after the first treatment, and 2 months after the completion of the first cycle of treatment.

### Response to treatment

All patients underwent a baseline four-phase contrast enhanced CT 1 day before the treatment. Treatment response was evaluated based on follow-up contrast enhanced CT according to modified Response evaluation criteria in solid tumours (mRECIST) by a randomly assigned experienced abdominal and/or interventional radiologists.^18^ The first follow-up CT for the evaluation of treatment response was scheduled 2 months after completing the first cycle of treatment. Depending on the disease burden, the first treatment cycle consisted of 1, 2 or 3 procedures to ensure complete coverage of tumours before the first treatment response evaluation. An objective response (OR) to treatment was reported in patients achieving a complete response (CR) or a partial response (PR). In cases of OR, follow-up CT was scheduled every 3 months the first year, after that, follow-up imaging was scheduled every 6 months or until progression of disease (PD). Every patient achieving stable disease (SD) or PD was re-evaluated by the MTB for further treatment strategies.

### Pharmacokinetic study

For the pharmacokinetic study, plasma levels of idarubicin and its major metabolite idarubicinol were measured at baseline (*i.e.* before the first procedure) and at 5, 15, 30 minutes, 2, 6, 10, 24, and 72 hours after the first procedure. All samples were obtained from peripheral venous blood and were immediately centrifuged upon collection. After centrifugation, the plasma samples were extracted by tert-buthylmethyl ether with 10% isopropanol and then subjected to analysis by a high-performance liquid chromatograph Agilent 1290 Infinity II (Waldbronn, Germany), coupled to a hybrid triple quadrupole/ion trap mass spectrometer Sciex 5500 Qtrap (Framingham, MA, USA). The method was validated in terms of selectivity, accuracy, precision, recovery, relative matrix effect, stability, and sensitivity with the lower limit of quantitation at 0.025 ng/mL for both idarubicin and idarubicinol. The mean peak plasma concentration (C_max_) and mean time to peak plasma concentration (T_max_) of idarubicin and idarubicinol were determined, moreover the mean area under the concentration curves from 0 to 72 hours (AUC_0-72_) was calculated using the trapezoidal method.

### Statistical analysis

Categorical variables were expressed using frequencies and percentages. Continuous variables were expressed as the mean and standard deviation (SD), in case of skewed distributions, median and range were used.

Progression free survival (PFS) was defined as the time from the first TACE procedure until initial progression, either after which patient remained a candidate for retreatment with TACE or underwent discontinuation of TACE from all causes (including death). Time to TACE untreatable progression (TTUP) was defined as the time from the first TACE procedure until discontinuation of TACE after radiological and/or clinical progression that prevented retreatment with TACE. Median PFS and TTUP were calculated using the Kaplan-Meier method.

Median follow-up was calculated from the day of the first TACE until either death or the end of the study using the reverse Kaplan-Meier method. Survival was calculated from the day of the first TACE until death, surviving patients were censored at the end of the study. Survival curves were determined using the Kaplan-Meier method. To assess potential associations between pharmacokinetic parameters and response to treatment or other clinical outcomes, non-parametric Mann-Whitney U tests and logistic regression models were conducted, respectively. *p*-values < 0.05 were considered statistically significant. Additionally, receiver operating characteristic (ROC) curves were plotted to evaluate the prognostic value of idarubicin/idarubicinol plasma concentrations and to identify optimal cut-off values.

The statistical analyses were performed using IBM^®^ SPSS^®^ Statistics Version 29.0.2.0. (International Business Machines Corporation, Armonk, New York, USA) for macOS.

## Results

### Patients

The baseline demographic, clinical, laboratory and imaging characteristic of 31 intermediate stage HCC patients who were enrolled to present study are summarized in Table 1. Most patients were male (90.3%). The mean patient age was 70.6 ± 6.7 years. Most patients (96.8%), except one, presented with cirrhotic liver, of them 67.7% of ethylic aetiology. The second most common cause of cirrhosis was non-alcoholic steatohepatitis (NASH). Cirrhosis was classified as Child-Pugh A and B in 23 (76.7%) and 7 (23.3%) patients, respectively. Clinical signs of portal hypertension and ascites were observed in 21(67.7%) and 10 (32.3%) patients, respectively. Baseline median serum AFP was 5,3 kU/L (range: 1-4062.5). Sixteen (51.6%) patients presented with unilobar disease. The mean number of lesions per patient was 3.1 ± 2.1 and the mean diameter of largest lesion was 44.8 ± 22.9 mm. The mean baseline LVEF was 64.1 ± 8.7 %.

**TABLE 1.** Baseline patient's demographic, clinical, laboratory and imaging characteristics

**Sex, number of patients (%)**	
Male/female	28 (90.3)/3 (9.7)
**Age, years**	
Mean ± SD	70.6 ± 6.7
**Cirrhosis, n. (%)**	
Yes/No	30 (96.8)/1 (3.2)
**Cirrhosis aetiology, n. (%)**	
Ethylic	21 (67.7)
NASH	4 (12.9)
Hemochromatosis	2 (6.5)
HBV	1 (3,2)
HCV	1 (3,2)
Cryptogenic	1 (3,2)
**Portal hypertension, n. (%)**	
Yes/No	21 (67.7)/10 (32.3)
**Ascites, n. (%)**	
Yes/No	10 (32.3)/21(67.7)
**Laboratory characteristics**	
GGT, median (range) [μkat/L]	1.72 (0.24–14.83)
AST, mean ± SD [μkat/L]	0.80 ± 0.26
ALT, mean ± SD [μkat/L]	0.71 ± 0.39
Total bilirubin, mean ± SD [μmol/L]	25.03 ± 15.9
Albumin, mean ± SD [g/L]	40.90 ± 5.00
AFP, median (range) [kU/L]	5.3 (1–4062.5)
**Child-Pugh score (n = 30)**	
Mean points ± SD	5.8 ± 1.0
**Child-Pugh class (n = 30)**	
A/B, n (%)	23 (76.7)/7 (23.3)
**Imaging characteristics**	
Number of lesions, mean ± SD [mm]	3.1 ± 2.1
Diameter of largest lesion, mean ± SD [mm]	44.8 ± 23.0
Cumulative diameter of lesions, mean ± SD [mm]	75.7 ± 39.8
Unilobar disease, n (%)	16 (51.6)
Bilobar disease, n (%)	15 (48.4)
**LVEF (%)**	
Mean ± SD	64.1 ± 8.7

AFP = alpha fetoprotein; ALT = alanine aminotransferase; AST = aspartate aminotransferase; GGT = gamma-glutamyl transferase; HBV = hepatitis B virus; HCV = hepatitis C virus; LVEF = left ventricular ejection fraction; NASH = non-alcoholic steatohepatitis

### Procedures

Overall, 68 DEMIDA-TACE procedures were performed. Depending on the disease burden, patients underwent 1 (3.2%), 2 (74.2%) or 3 (22.6%) procedures before the first efficacy evaluation.

### Adverse events

Overall, AEs grade ≥ 3 were noted after 20 (29.4%) procedures in 13 (41.9%) patients. AE grade 3 elevation of AST, GGT, ALT and bilirubin was observed in 7 (10.3%), 4 (5.9%), 4 (5.9%), and 3 (4.4%) procedures, respectively. While the laboratory changes observed were transient, they persisted up to 72 hours after the procedure. Notably, all but 1 patient, who presented with grade 3 elevation of GGT, returned to their pre-treatment laboratory status at 14 days follow-up after the first procedure.

Grade 3 abdominal pain was noted in 7 (10.3%) of procedures. No grade ≥ 3 haematologic and cardiotoxicity was observed. Three (in 4.4% of all procedures) major complications were noted: a pseudoaneurysm formation at the puncture site, which was treated by endovascular embolization with coils; and an infection of necrotic target lesion with abscess formation, necessitating percutaneous drainage and antibiotic treatment in two patients. Postembolization syndrome (*i.e.* abdominal pain, fever, nausea, elevation (doubling of baseline value) of liver enzymes, leucocytosis) was observed after 10 (14.7%) procedures.

### Response to treatment

**FIGURE 1.**
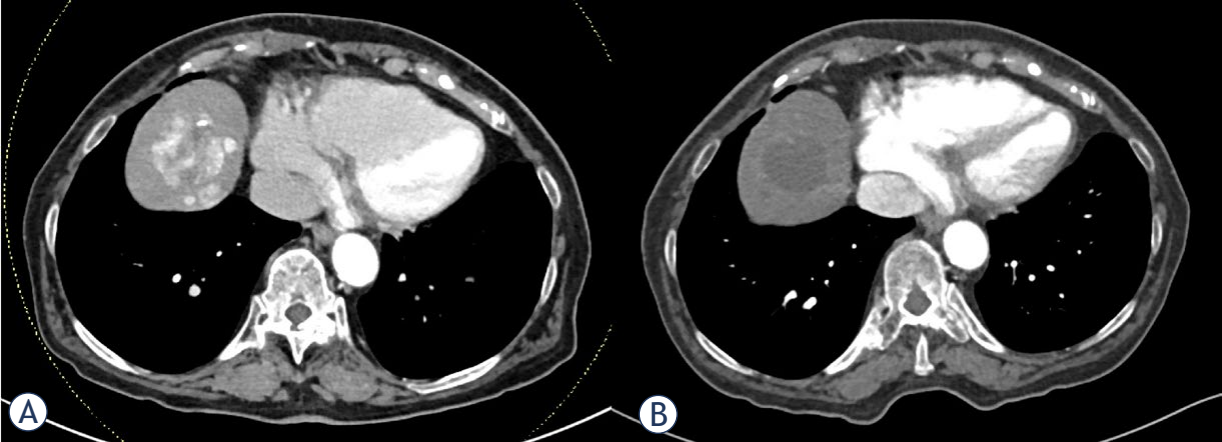
CT slices showing a mRECIST partial response to idarubicin-loaded drug-eluting microspheres (DEMIDA) - transarterial chemoembolization (TACE) in the VIII. segment of cirrhotic liver. **(A)** Baseline CT showing multifocal hepatocellular carcinoma (HCC). The diameter of the largest tumour was 38 mm; **(B)** Only minimally enhancing largest target lesion at the first treatment response evaluation, the diameter of viable lesion was 4 mm.

At the first follow-up imaging, an OR in target lesions was achieved in 29 patients (93.5%). An overall OR was achieved in 26 (83.9%) patients (Figure 1). Overall CR and PR were achieved in 9 (29% of all 31 patients) and 17 (54.8%) patients, respectively. Three patients achieved PR in target lesions, however overall TACE treatable PD due to development of new lesions. Two patients presented with clinical and radiological (PD in target lesions as well as development of new lesions) TACE untreatable progression: first patient died 5.4 months after the first procedure due to progression to macrovascular invasion of the middle hepatic vein and inferior vena cava; second patient with intrahepatic progression and decompensation of liver cirrhosis died 6.5 months after the first procedure.

During the follow-up, progression (or death) was observed in 30 (96.8%) patients. The median PFS was 10.5 months (95% CI: 6.8–14.3 months). Thirteen patients (43.3% of patients with progression) underwent additional on-demand TACE treatment. Five (16.7%) patients received systemic therapy, while 8 (27%) received best supportive care. One (3.3%) patient underwent surgical resection. During the follow-up, TACE untreatable progression was observed in 18 (58,1%) patients. The median TTUP was 24.6 months (95% CI: 11.6–37.6 months).

### Survival

**FIGURE 2.**
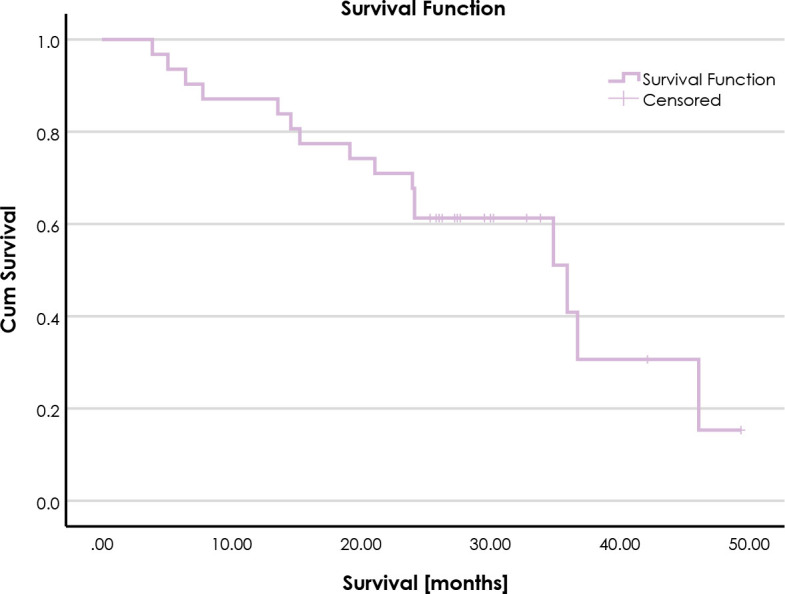
Kaplan-Meier curve for overall survival.

During the median follow-up of 30 months (95% CI 29.0–31.0 months), 16 patients died. The 1- and 2-year survival rates were 87% and 71%, respectively. Median OS was 36.0 months (95% CI 21.1–50.9 months) (Figure 2).

### Idarubicin pharmacokinetics

**FIGURE 3.**
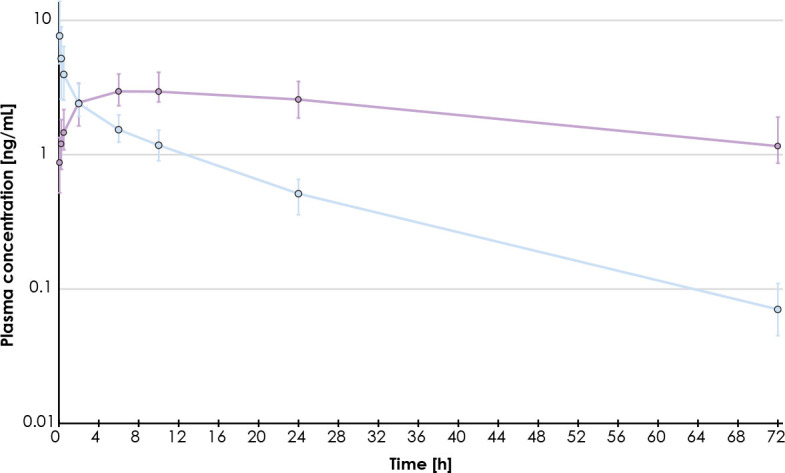
Geometric mean plasma concentration profiles for idarubicin (blue) and idarubicinol (purple) (n = 31). Error bars indicate first and third quartiles of plasma concentrations.

The idarubicin and idarubicinol plasma concentrations within the initial 72-hour period following DEMIDA-TACE remained low, with the mean C_max_ of 9.1 ± 5.0 ng/mL and 3.7 ± 1.6 ng/mL, respectively (Figure 3). The median T_max_ for idarubicin was 5 min (range, 5–15 min), indicating rapid systemic absorption following hepatic intra-arterial administration. In contrast, idarubicinol plasma concentrations exhibited a slower increase, with a median T_max_ of 10 hours (range, 2–24 hours). However, the mean AUC_0-72_ for idarubicinol was higher at 179.7 ± 81.4 ng/mL*h compared to idarubicin (mean AUC_0-72_ 54.0 ± 25.8 ng/mL*h) over the same 72-hour period, indicating a greater overall exposure to the metabolite rather than the parent drug.

**FIGURE 4.**
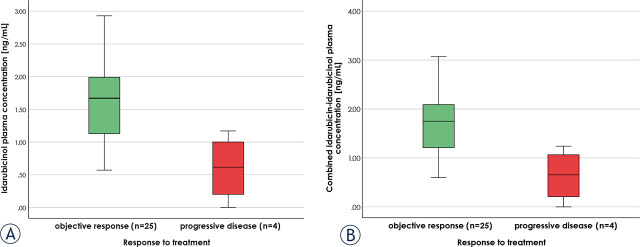
Idarubicinol **(A)** and combined idarubicin-idarubicinol **(B)** plasma concentrations at 72 hours post-procedure in patients with objective response to treatment or progressive disease (n = 29).

Patients achieving OR had significantly higher concentrations at 72 hours for both idarubicin and idarubicinol (p = 0.024 and 0.014, respectively), as well as their combined concentration (p = 0.014), compared to those with PD. The cut-off values for OR were identified at 1.2 ng/mL and 1.29 ng/mL for idarubicinol and combined idarubicin-idarubicinol plasma concentration, respectively (Figure 4). The sensitivity and specificity of both cut-off values were 0.72 and 1, respectively. Median idarubicinol and combined idarubicin-idarubicinol plasma concentration at 72 hours were 1.62 ng/mL (range, BLOQ–2.93 ng/mL) and 1.72 ng/mL (range, BLOQ–3.07 ng/mL), respectively. However, in terms of AUC_0-72_ for idarubicin, idarubicinol and their combined plasma concentration, no significant differences were observed between OR and PD groups.

Furthermore, no significant differences were observed in pharmacokinetic parameters between patients experiencing AEs grade ≥ 3 and those without such events.

## Discussion

The present prospective study aimed to evaluate safety, efficacy, and pharmacokinetics of DEMIDA-TACE for the intermediate stage HCC patients. The promising outcomes of present study suggest that this approach to treatment holds significant potential in the management of HCC.

To our knowledge, our study is the largest in terms of the study population treated with DEMIDA-TACE using LiferPearl™ microspheres. The mechanical and pharmacological properties of LiferPearl™ microspheres have been studied *in vitro* and compared with four commonly used microspheres (LifePearl, DC Bead, HepaSphere, and Tandem).^19^ The PARIS registry study enrolled 97 patients including 22 patients treated with idarubicin loaded DEM and 75 patients treated with doxorubicin loaded DEM.^20^

In our study population, AEs grade ≥ 3 were noted in 41.9% patients after 29.4% procedures, including postembolization syndrome observed after 10 (14.7%) procedures. The PARIS registry using LiferPearl™ microspheres loaded with either idarubicin or doxorubicin reported AEs grade ≥ 3 in 13.4% patients, mainly related to postembolization syndrome. Moreover, the PARIS registry reported no AEs grade ≥ 3 laboratory changes except one transient hyperbilirubinemia. However, one patient with a history of heart insufficiency died from heart failure two days after DEMIDA-TACE performed with 4 mL of LifePearl™ loaded with 5 mg/mL of idarubicin, without postembolization syndrome.^20^ When administrated intravenously, the main safety concern for idarubicin is dose-limiting cardio- and haematological toxicity. No cardiotoxicity was noted in our study population, consistent with other research on DEMIDA-TACE.^14,15,20,21^ This can be explained by the cumulative cardiotoxic dose of idarubicin set at 93 mg/m^2^.^22^ Theoretically, a patient with an average body surface area of 1.8 m^2^ and normal LVEF can undergo 17 sessions of TACE using 10 mg of idarubicin before reaching this threshold. Furthermore, studies have shown that idarubicin-induced cardiomyopathy is uncommon with cumulative doses up to 290 mg/m^2^.^14,22^ No haematological toxicity was observed in our study population. In a phase I dose-escalation trial study of 21 patients treated with TANDEM (Boston Scientific, Marlborough, Massachusetts) microspheres, half of haematological grade 3 AEs observed were thrombocytopenia, which occurred in patients with a baseline platelet count < 75 000 per mmc.^14^ In a study of 72 patients by the same institution, grade 3–4 AEs after 52% of sessions, being primarily biological (such as elevation of AST, ALT, bilirubin, GGT, glucose, AP and lipase, in order of frequency), while clinical manifestations (such as abdominal pain, fatigue, fever and ascites) were observed to a lesser extent. Additionally, hepatobiliary complications were reported in 2 sessions, including gallbladder necrosis in one patient and multi-organ failure leading to death in another.^22^ It is noteworthy that in PARIS registry there were no significant differences in the rate of hepatobiliary toxicities between idarubicin (10/31, 9.7%) and doxorubicin (26/156, 16.7%; p = 0.58) groups. However, there was a trend towards less hepatobiliary toxicities in the idarubicin group, despite significantly lower number of patients with cirrhosis, which has a protective role against hepatobiliary complications.^20,23,24^ Of 3 major complications (4,4% of all procedures) in the present study, pseudoaneurysm at puncture site cannot be reliably attributed to the use of idarubicin. Four major complications have been reported out of 452 TACE (0.9%) in 144 patients, that were treated with DEMDOX-TACE at our institution between February 2010 and December 2018. An ischemic cerebrovascular insult to the cerebellum following a non-target embolization (the tumour feeding artery being the right internal mammary artery); a radial artery thrombosis following a trans radial approach; a variceal bleeding resulting from emesis after the procedure; and an infection of the necrotic tumour, which resolved after antibiotic treatment.^25^ Those complications, as well as major complications in the present study, were not related to the specific chemotherapeutic agent. The hepatic intraarterial administration of idarubicin-loaded drug-eluting microspheres allows maximizing drug exposure in the targeted tumour while minimizing systemic exposure and potential systemic toxicity, suggesting that DEMIDA-TACE holds significant potential for optimizing therapeutic outcomes while minimizing AE.

The ORR of 83.9% within 2 months after first cycle of DEMIDA-TACE (including 29.5% CR) in our study is in line with the best previously reported OR rate for LifePearl™ microspheres ranging between 76% and 90.9%.^26,27,28,29,30^ The reported OR at the first follow-up imaging in our group of patients treated with DEMDOX-TACE was 91.0%. Additionally, CR rate achieved in those patients was higher than in our idarubicin group, 55.0% *vs*. 29.5%, respectively.^25^ PARIS registry reported an 81% ORR in target lesions and 72.9% overall ORR.^20^ The best reported ORR by Guiu *et al.* was 65% (61% after the first session, 64% after the second, 61% after the third, 50% after the forth session).^22^ High ORR observed in patients treated at our institution, using doxorubicin or idarubicin, are likely attributable to the regular superselective approach using microcatheter and repeated CBCT for depicting lesions and feeding arteries within the same session. While this approach prolongs procedures, it also ensures superselective delivery to each target lesion, likely resulting in higher OR.^25,31^

Our reported median OS of 36.0 months (95% CI 21.1–50.9 months) is in line with the reported median OS in patients treated with TACE, which ranges from less than 20 months in real life cohorts, up to 45 months in well-selected patients.^32^ According to the BCLC 2022 update, the expected survival of patients treated with TACE is more than 30 months.^4^ The reported median OS in our DEMDOX-TACE group was 25.8 months. The 1- and 2-year survival rates in those patients were 85% and 53%, respectively, whereas in our idarubicin group, 1- and 2-year survival rates were 87% and 71%, respectively.^25^

Today, the reported OS results not only from DEM-TACE, but also from additional and subsequent treatment strategies, including systemic therapies that have highly improved during the past years namely due to the combined immune- and VEGF inhibitors therapy. PFS and TTUP were introduced as secondary endpoints, as they serve as a surrogate marker of OS to minimize the impact of other treatment strategies.^3,33,34^ The median PFS and median TTUP of 10.5 months and 24.6 months, respectively, are the longest reported in patients treated with DEMIDA-TACE. The PARIS registry reported median PFS of 10.4 and 15.7 months in their idarubicin and doxorubicin group, respectively, with a shorter median PFS in the idarubicin group being explained by the larger size of treated tumours in this group.^20^ In our DEMDOX-TACE experience, the reported median PFS was 10.2 months, comparable with the present idarubicin group.^25^ Guiu *et al.* reported median PFS of 6.6 months in their IDASPHERE II trial and time to treatment failure (time until TACE discontinuation due to any reason, including death) of 14.4 months in patients treated with idarubicin-loaded TANDEM beads (Boston Scientific, Marlborough, Massachusetts).^15,21^

The plasma concentrations of both idarubicin and idarubicinol within the 72-hour post DEM-TACE remained low, demonstrating the ability of DEM to release idarubicin in a sustained manner. The median T_max_ of 5 minutes suggests that idarubicin reaches its maximum systemic concentration relatively quickly after hepatic intraarterial administration, consistent with the results of IDASPHERE phase 1 trial, thus demonstrating an initial moderate burst release. In contrast, plasma concentrations of idarubicinol increased slowly; with a median T_max_ of 10 hours even slower than with DC Bead™ (Biocompatibles, Surrey, UK) microspheres, for which Boulin *et al.* reported a median T_max_ of 6 hours (range 2–6 hours). However, the mean AUC_0-72_ results of 54.0 ± 25.8 ng/mL*h and 179.7 ± 81.4 ng/mL*h, for idarubicin and idarubicinol, respectively) are closer to those of the 15 mg dose study group from IDASPHERE I, for which values of mean AUC_0-72_ were reported as 146.8 ± 134.9 ng/mL*h and 256.0 ± 79.9 ng/mL*h, respectively. This may be attributable to various factors: a higher lower limit of quantification in IDASPHERE study (1 ng/mL *vs.* 0.025 ng/mL) as well as use of different brand and sizes of microspheres (DC Bead™ of 300–500 μm v*s.* LifePearl 100 μm), as indeed for the same volume of microspheres, smaller microspheres size have a larger number of microspheres resulting in more contact surface likely resulting in faster and higher elution.^14^

The higher mean AUC_0-72_ for idarubicinol compared to idarubicin indicates a greater overall exposure to the metabolite rather than the parent drug. The significant difference observed in idarubicin, idarubicinol, and their combined plasma concentrations at 72 hours between patients achieving OR and those with PD suggests that pharmacokinetics may serve as predictive factor for treatment outcomes. In patients with idarubicinol and combined idarubicin-idarubicinol plasma concentrations at 72 hours post-procedure above the defined cut-off values (1.2 and 1.29 ng/mL, respectively), achieving OR to treatment is associated with a 0.72 sensitivity and 1.0 specificity. Further investigations with larger sample sizes are warranted to confirm these findings.

Limitations of present study include a single institution setting with a small study population and no control group treated with DEMDOX-TACE. However, the results of present study were compared to those of our previously published retrospective study involving 144 consecutive patients treated with DEMDOX-TACE at our institution.^25^

In conclusion, our study demonstrates promising results regarding the safety, efficacy, and pharmacokinetics of DEMIDA-TACE in intermediate stage HCC patients. Lifepearl™ microspheres efficient delivery of idarubicin to the targeted lesions resulting in minimal systemic toxicity, a high ORR, prolonged PFS and TTUP, and favourable OS, support the use of idarubicin as a viable alternative to doxorubicin in DEM-TACE. Further multicentric randomized trials are warranted to objectively assess the potential superiority of idarubicin over doxorubicin and other commonly used anticancer drugs in this setting.

Idarubicinol and combined idarubicin-idarubicinol plasma concentrations at 72 hours post-procedure may serve as prognostic factors for achieving OR.

## Supplementary Material

Supplementary Material Details
