# Supplementary material for: Idarubicin-loaded drug-eluting microspheres transarterial chemoembolization for intermediate stage hepatocellular carcinoma: safety, efficacy, and pharmacokinetics
Source: Radiol Oncol. 2024 Oct 4;58(4):517–26. doi: 10.2478/raon-2024-0052 (PMC11702893; doi:10.2478/raon-2024-0052)
Supplement: Supplementary file 1 — Supplementary Material Details [file raon-2024-0052_sm.pdf]

sciendo
Radiology and Oncology | Ljubljana | Slovenia | www.radioloncol.com
RADIOLOGY AND ONCOLOGY

Idarubicin-loaded drug-eluting microspheres transarterial chemoembolization for intermediate stage hepatocellular carcinoma: safety, efficacy, and pharmacokinetics

Spela Korsic, Josko Osredkar, Alojz Smid, Klemen Steblovnik, Mark Popovic, Igor Locatelli, Jurij Trontelj, Peter Popovic

doi: 10.2478/raon-2024-0052

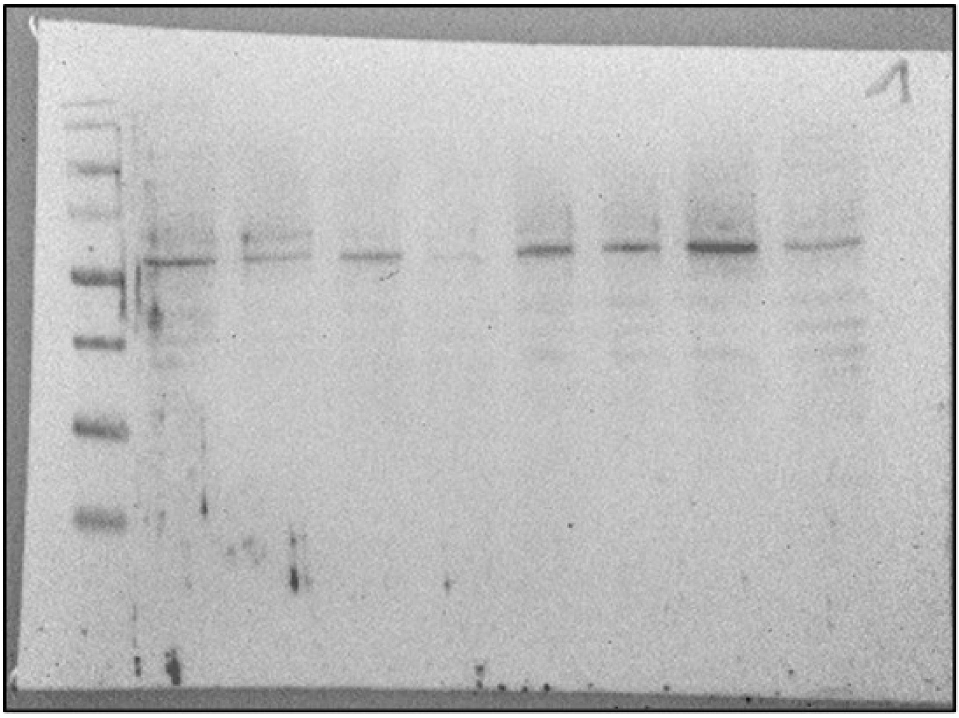

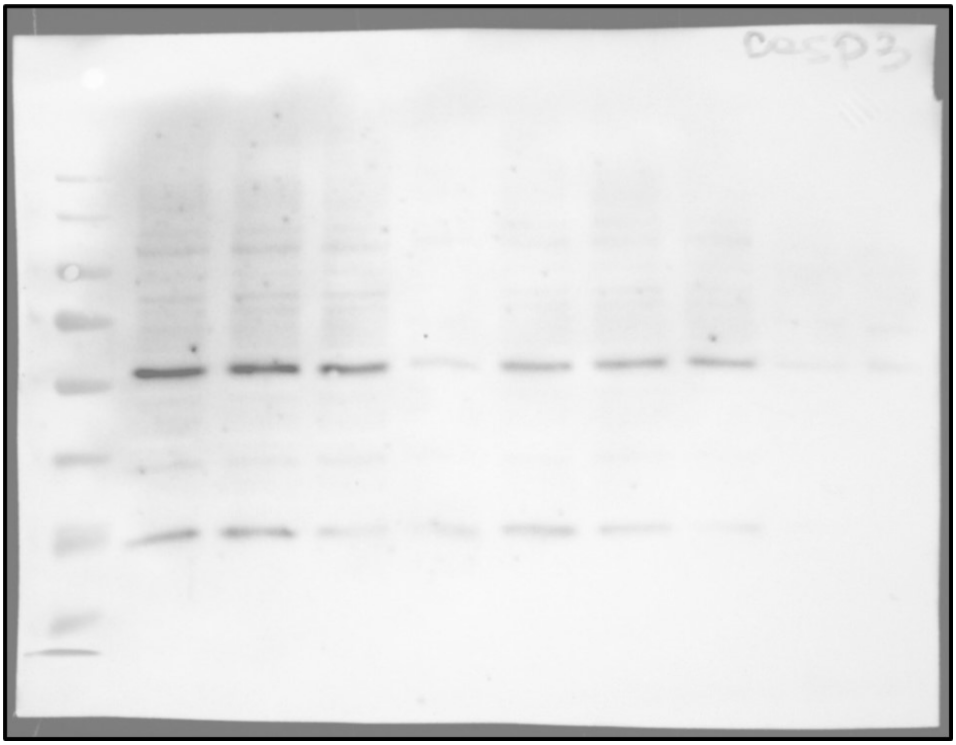

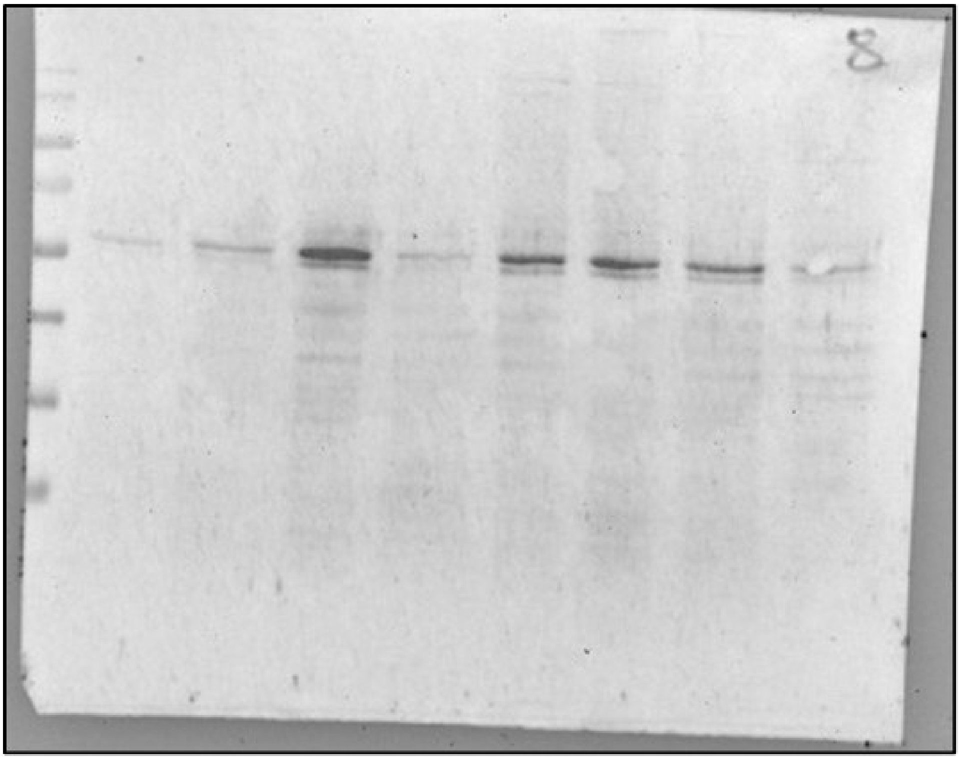
8

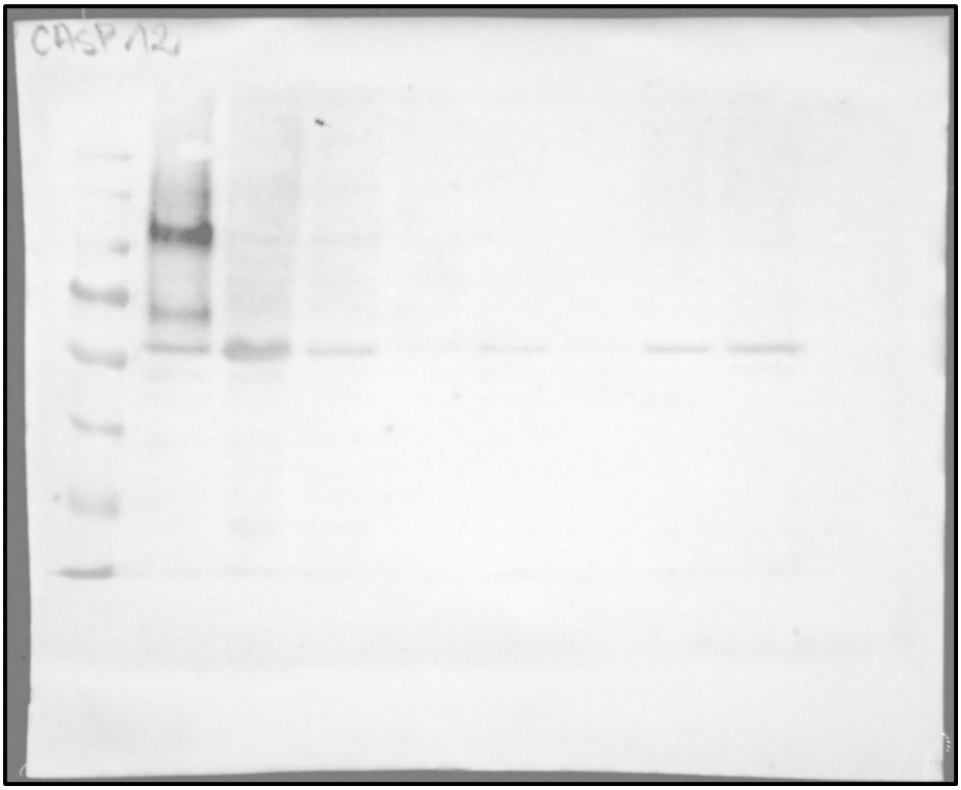
CASP12

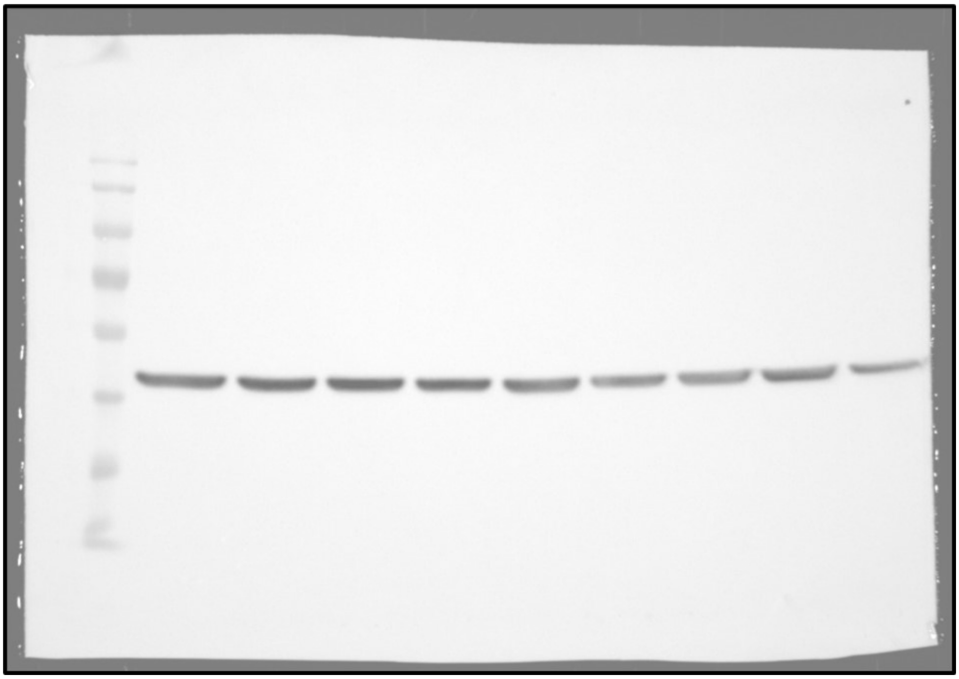

FIGURE 1 Original blots images of *(from the top)*: caspase-1; caspase-3; caspase-8; capsase-12 and β-actin.
